# Maternal, obstetric and gynecological factors associated with preterm birth in Rwanda: findings from a national longitudinal study

**DOI:** 10.1186/s12884-023-05653-y

**Published:** 2023-05-19

**Authors:** Erigene Rutayisire, Monica Mochama, Corneille Killy Ntihabose, Jean Nepo Utumatwishima, Michael Habtu

**Affiliations:** 1 College of Medicine and Health Sciences, School of Public Health, University of Rwanda, Kigali, Rwanda; 2 Public Health Department, Mount Kenya University, Kigali, Rwanda; 3 Department of Clinical and Public Health Services, Ministry of Health, Kigali, Rwanda; 4 Rwamagana Level Two Teaching Hospital, Ministry of Health, Kigali, Rwanda; 5 Sahlgrenska Academy, University of Gothenburg, Gothenburg, Sweden

**Keywords:** First-trimester, Longitudinal cohort study, Preterm birth, Risk factors

## Abstract

**Background:**

Preterm birth is one of the key causes of morbidity and mortality among neonates in low-income countries. In Rwanda, at least 35,000 babies are born prematurely each year, and 2600 children under the age of five die due to direct complications of prematurity each year. A limited number of studies have been conducted locally, many of which are not nationally representative. Thus, this study determined the prevalence as well as the maternal, obstetric, and gynecological factors associated with preterm birth in Rwanda at the national level.

**Methods:**

A longitudinal cohort study was conducted from July 2020 to July 2021 among first-trimester pregnant women. A total of 817 women from 30 health facilities in 10 districts were included in the analysis. A pre-tested questionnaire was used to collect data. In addition, medical records were reviewed to extract relevant data. Ultrasound examination was used to assess and confirm gestational age on recruitment. A multivariable logistic regression analysis was performed to determine the independent maternal, obstetric, and gynecological factors associated with preterm birth.

**Results:**

The prevalence of preterm births was 13.8%. Older maternal age- 35 to 49 years [Adjusted odds ratio (AOR) = 2.00; 95% Confidence Interval (CI) = 1.13–3.53)], secondhand smoke exposure during pregnancy (AOR = 1.91; 95% CI = 1.04–3.51), a history of abortion (AOR = 1.89; 95% CI = 1.13–3.15), premature membrane rupture (AOR = 9.30; 95% CI = 3.18–27.16), and hypertension during pregnancy (AOR = 4.40; 95% CI = 1.18–16.42) were identified as independent risk factors for preterm birth.

**Conclusion:**

Preterm birth remains a significant public health issue in Rwanda. The associated risk factors for preterm birth were advanced maternal age, secondhand smoke, hypertension, history of abortion, and preterm membrane rupture. This study therefore recommends routine antenatal screening to identify and closely follow-up of those high-risk groups, in order to avoid the short- and long-term effects of preterm birth.

## Background

Preterm birth is the delivery of the fetus before 37 weeks of gestation. According to estimates from the World Health Organization, 15 million (or one in ten) babies are born early each year [1]. African and South Asian countries account for more than 80% of these preterm births [2]. On average, 12% of babies are born prematurely in low-income nations, compared to 9% in high-income countries [3] with differences in incidence among countries. According to different studies, this incidence is reported to vary from 5.0% in Sweden [4], 16.8% in Nigeria [5], 18.3% in Kenya [6], and between 4.4 and 25.9% in Ethiopia [7–10].

Preterm birth is the primary cause of perinatal illness and mortality worldwide [2]. It is one of the biggest healthcare challenges as it is associated with long-term disability and financial strain from the costs of care especially in underdeveloped nations [11]. According to Wagura et al. [6], it accounts for around one-third of all neonatal deaths as a result of increased risk of infection [12]. It also has long-term adverse effects, such as poor neurodevelopment leading to learning disabilities, cerebral palsy, and vision abnormalities among others [13].

Studies have found a number of risk factors for preterm birth, which can be categorized as: (1) Maternal risk factors, such as maternal age, education level, low socioeconomic status, marital status, maternal malnutrition, substance use, inadequate antenatal care (ANC), and stress, among others [14–17], (2) gynecological risk factors such as intrauterine infection, urinary tract infection, pregnancy induced hypertension, sexually transmitted infections, premature rupture of membranes, uterine malformations, and uterine adhesions [18–21] and (3) Obstetric risk factors include: primiparity, short birth interval, antenatal care, multiparity, history of preterm birth, history of abortion, history of stillbirth or miscarriage, and multiple pregnancies [22–24]. However, the factors from various studies are uneven, with some factors showing direct association in some studies but having an inverse association or no association in others.

In Rwanda, despite the improvement of maternal and child health services, at least 35,000 babies are born too soon each year, and 2600 children under five die due to direct preterm complications [25]. The factors causing increased preterm birth in Rwanda are currently unknown. Some small-scale studies representing specific locality or hospital have been done. For instance, Nwankwo et al. demonstrated preterm birth prevalence of 17.5% with husband’s smoking, low ANC attendance and low maternal MUAC as independent predictors of preterm birth [26]. This was a single center facility based cross-sectional study whose findings cannot be generalized. However, as far as we are aware, there is no publication of a longitudinal study that attempt to investigate risk factors associated with preterm birth at a national level. Hence, this study aimed at determining the maternal, obstetric, and gynecological factors associated with preterm birth in Rwanda.

## Methods

### Study design and setting

Rwanda is a landlocked country located in central-eastern part of Africa. It is a low-income country with a population of about 13 million people. The country ‘health system is a pyramid with the top of pyramid, being the Ministry of health responsible for sector coordination and oversight, and setting health policies and strategies; Rwanda Biomedical Centre responsible of implementation of health programs such as Tuberculosis, Malaria, HIV and Maternal and child health; Rwanda Food and Drugs Authority for regulation of human and veterinary medicines, vaccines and other biological products, processed food, poisons, medical cosmetics, medical devices, and other products; and Rwanda Medical Supply with the mandate of ensuring availability of medicines, medical supplies and consumables to the health facilities. Currently, Rwanda has 565 public health facilities including 504 Health centers, 7 Medicalised Health Centers, 40 District, 4 Provincial, 4 Specialized and 8 Referral hospitals. Health posts are entities working at lowest level and operating under public-private partnership models; there are 1222 Health Posts (HPs) in Rwanda. Private health facilities are 317 and distributed as follow: 115 private dispensaries, 113 general clinics, 33 specialized clinics, 28 polyclinics, 9 dental clinics, 6 nutrition cabinets, 4 Private Hospitals, 4 Specialized hospitals, 3 Psychology clinics, and 2 laboratories [27].

A health facility follow-up study using a longitudinal single baseline cohort design among first-trimester pregnant women was conducted from July 2020 to July 2021. The study was conducted in 30 health facilities including hospitals and health centers located in 10 districts around the country. The sampling unit was hospital and then convenience sampling was used to select one health center in urban area and one health center in rural area under each selected hospital. Therefore, pregnant women in the first trimester from 10 hospitals and 20 health centers were recruited in the study.

### Population, sample size and sampling technique

Participants in this study were pregnant women in their first trimester. Being in the first trimester of pregnancy (within the 13 weeks gestation), intending to stay in the study for the duration of the study, and being able to sign or thumbprint informed consent were the inclusion criteria. However, pregnant women with chronic diseases such as hypertension, heart disease, and renal disease were excluded.

**Fig. 1:**
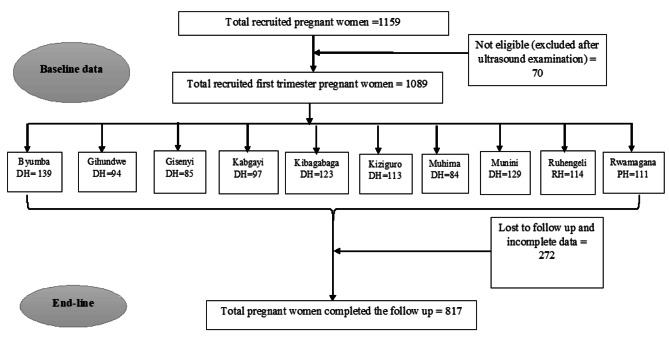
Flowchart displaying participant enrollment and follow-up

A multi-stage sampling strategy was adopted, with the first stage consisting of the simple random selection of 30 health facilities (acting as clusters) from around the nation (Fig. 1). Then, pregnant women who attended the antenatal care department in the selected healthcare facilities and were determined to be in their first trimester were included in the study. Between July and December 2020, all eligible women from each selected health facility were enrolled in the study. A total of 1159 pregnant women in their first trimester took part in the study after taking into account all inclusion criteria and 817 pregnant women were included in the analysis (Fig. 1).

### Data collection procedure and tools

30 qualified nurses and midwives collected the data under the direct supervision of 10 team leaders from Mount Kenya University, and the study team. Team leaders, nurses and midwives received one-day training on the study protocol, inclusion criteria, sampling, and research tools, and data collection procedures. The questionnaire was pretested among 50 pregnant women who attended two health centers in Kigali City for ANC services. With the results of this pilot study, the study tool was adjusted as necessary. Pregnant women were told about the goal of the study during the data collection procedure, and they consented to participate.

Face-to-face interviews were used to gather the data using a structured questionnaire, and antenatal care records were examined to gather some additional medical information on maternal conditions. The questionnaire was adopted from the literature and contextualized according to Rwandan context. The questionnaire was initially prepared in English, and then translated into Kinyarwanda, then back into English. At the base line (first trimester) as well as in the end line (shortly after delivery) factors related to maternal characteristics (age, residence marital status, level of education, employment, socio-economic status, alcohol use and smoking during pregnancy and exposure to passive smoking), obstetric (parity, birth spacing, mode of delivery ANC use, preterm rupture of membrane, amniotic fluid volume HIV status Blood group and history of still birth, abortion and previous Cesarean section) and gynecologic (hospitalization during pregnancy, gestational diabetes, hypertension in pregnancy, malaria during pregnancy, hepatitis B, urethritis infection, cystitis infection, pyelonephritis infection and vaginitis infection) were collected.

#### Measurement of the primary outcome (pre-term birth)

Preterm delivery, which is defined as a live birth at less than 37 weeks of gestation, was the main outcome in this study. The latest menstrual period (LMP) date was used to estimate the gestational age, and an ultrasound examination was used to confirm this. Since ultrasounds are not typically performed at health centers, the pregnant women recruited from the health centers were directed to the nearest hospital for ultrasound examination. Ultrasound examination was done by the obstetrician or general practitioner at hospital.

### Data analysis

Using IBM SPSS Statistics 25, data was entered and examined. Counts and percentages were used to describe the characteristics of respondents. Tables and text were used to present the results. For each categorical independent variable, a Chi-square assumption was made. Then, binary logistic regression using bivariate and multivariate analysis was used to identify the risk factors related to preterm birth. For the multivariable logistic regression analysis, variables having a p-value of less than 0.2 in the bivariate logistic regression were taken into account. The Adjusted Odds Ratio (AOR) with a 95% confidence interval was determined in the multivariable logistic regression analysis. Finally, factors were deemed significant if their p-value was less than 0.05. The Hosmer-Lemeshow test was used to gauge the model’s fitness.

## Ethical consideration

With reference No. 131/RNEC/2020, the Rwanda National Ethics Committee granted its approval, and each of the chosen hospitals provided a letter of permission. Participants in the study were given a thorough description of the study’s objectives before providing their written informed consent. Legally authorized representatives of illiterate participants provided informed consent for the study. Informed consent was obtained from a parent and/or legal guardian for participants aged under 16.

Participants in the study had the freedom to decline or leave at any moment without incurring any penalties. Furthermore, the participants received assurances regarding the confidentiality of the data, and no personal identifying information was included on the questionnaires.

## Results

### Socio-demographic and lifestyle factors stratified by preterm

A total of 817 women and new-born pairs were included in the study to achieve the objectives. The prevalence of preterm birth was 13.83%, with a 95% confidence interval of 11.54% and 16.39%. The majority of new-born babies (63.2%) were female; however, this was not significantly associated with preterm birth. Similarly, there was no statistically significant association observed between risk of preterm birth and residence, marital status, level of education, occupation, and social class, as well as alcohol consumption and smoking during pregnancy. However, a significant association was observed between maternal age, partners’ smoking status, and the risk of preterm birth. The proportion of mothers who delivered preterm babies was significantly higher in the 35–49 age group (23% vs. 13.4%, p = 0.018) (Table 1). Mothers with preterm deliveries were more likely to be exposed to secondhand smoke compared to mothers with full-term births (p = 0.017) (Table 1).

**Table 1:** Socio-demographic and lifestyle factors stratified by preterm

Attributes	Pre-term		Full-term		Total		p value
	n	%	n	%	n	%	
**Gender of the newborn baby**							
Male	36	31.9	265	37.6	301	36.8	0.237
Female	77	68.1	439	62.4	516	63.2	
**Maternal age [years]**							
15 to 24	44	38.9	340	48.3	384	47.0	**0.018**
25 to 34	43	38.1	270	38.4	313	38.3	
35 to 49	26	23.0	94	13.4	120	14.7	
**Residence**							
Rural	84	74.3	525	74.6	609	74.5	0.957
Urban	29	25.7	179	25.4	208	25.5	
**Maternal marital status**							
Married	62	54.9	307	43.6	369	45.2	0.082
Cohabiting	43	38.1	338	48.0	381	46.6	
Single	8	7.1	59	8.4	67	8.2	
**Maternal level of education**							
No formal education	8	7.1	64	9.1	72	8.8	0.410
Primary	64	56.6	438	62.2	502	61.4	
Secondary	37	32.7	180	25.6	217	26.6	
Tertiary	4	3.5	22	3.1	26	3.2	
**Occupation**							
Government employed	8	7.1	49	7.0	57	7.0	0.404
Business	14	12.4	87	12.4	101	12.4	
Farmer	72	63.7	392	55.7	464	56.8	
House wife	16	14.2	155	22.0	171	20.9	
Others	3	2.7	21	3.0	24	2.9	
**Socio-class category**							
Social category 1^x^	21	18.6	116	16.5	137	16.8	0.678
Social category 2^y^	57	50.4	386	54.8	443	54.2	
Social category 3^z^	35	31.0	202	28.7	237	29.0	
**Maternal alcohol use during pregnancy**							
Yes	27	23.9	147	20.9	174	21.3	0.468
No	86	76.1	557	79.1	643	78.7	
**Maternal smoking during pregnancy**							
Yes	2	1.8	3	0.4	5	0.6	0.089
No	111	98.2	701	99.6	812	99.4	
**Partner’s smoking status**							
Yes	17	15.0	57	8.1	74	9.1	**0.017**
No	96	85.0	647	91.9	743	90.9	

*xCategory 1: Citizens who were homeless, extremely vulnerable, and unable to provide for their basic needs.*

*yCategory 2: Citizens who could afford to eat once or twice a day but were unemployed and could only afford some type of low-class owned or rented housing.*

*zCategory 3: Citizens who had a paying job or even employed people. Small farmers who went beyond subsistence farming or owners of small and medium-sized businesses were under this category.*

### Obstetric factors associated with preterm birth

The distribution of maternal obstetric factors according to gestational age is summarized in Table 2. Mothers with preterm deliveries attended fewer than four ANC visits than mothers with full-term deliveries (p = 0.021). Other obstetric factors associated with preterm birth that were statistically significant were history of abortion (p = 0.018), history of cesarean section (p = 0.042), and preterm membrane rupture (p < 0.001).

**Table 2:** Obstetric factors associated with preterm birth

Attributes	Pre-term		Full-term		Total		p value
	n	%	n	%	n	%	
**ANC visits**							
< 4	43	38.1	193	27.4	236	28.9	**0.021**
>=4	70	61.9	511	72.6	581	71.1	
**Parity**							
Primi-gravida	45	39.8	352	50.0	397	48.6	0.123
1 to 2	49	43.4	241	34.2	290	35.5	
2 to 3	15	13.3	73	10.4	88	10.8	
5+	4	3.5	38	5.4	42	5.1	
**Birth spacing (n = 389)**							
< 24	13	19.7	74	22.9	87	22.4	0.568
>=24	53	80.3	249	77.1	302	77.6	
**History of still birth**							
Yes	5	4.4	29	4.1	34	4.2	0.880
No	108	95.6	675	95.9	783	95.8	
**History of abortion**							
Yes	25	22.1	96	13.6	121	14.8	**0.018**
No	88	77.9	608	86.4	696	85.2	
**History of previous Cesarean section**							
Yes	16	14.2	58	8.2	74	9.1	**0.042**
No	97	85.8	646	91.8	743	90.9	
**Became pregnant while using contraceptive**							
Yes	11	9.7	45	6.4%	56	6.9	0.192
No	102	90.3	659	93.6%	761	93.1	
**Taking medicine in pregnancy**							
Yes	31	27.4	231	32.8%	262	32.1	0.255
No	82	72.6	473	67.2%	555	67.9	
**Preterm rupture of membrane**							
Yes	10	8.8	6	0.9	16	2.0	**< 0.001**
No	103	91.2	698	99.1	801	98.0	
**Amniotic fluid volume**							
Normal	105	92.9	663	94.2	768	94.0	0.602
Polyhydramnios	8	7.1	41	5.8	49	6.0	
**Mode of delivery**							
Normal	98	86.7	628	89.2	726	88.9	0.437
CS	15	13.3	76	10.8	91	11.1	
**Type of CS (n = 91)**							
Emergency	5	33.3	29	38.2	34	37.4	0.724
Elective	10	66.7	47	61.8	57	62.6	
**HIV status**							
Positive	2	1.8	17	2.4	19	2.3	0.673
Negative	111	98.2	687	97.6	798	97.7	
**Blood group**							
A	9	8.0	100	14.2	109	13.3	0.223
AB	3	2.7	25	3.6	28	3.4	
B	13	11.5	79	11.2	92	11.3	
O	31	27.4	215	30.5	246	30.1	
Not done	57	50.4	285	40.5	342	41.9	

### Gynecological factors associated with preterm birth

Table 3 presents factors related to gynecological conditions according to the gestational age at delivery. Hospitalization during pregnancy was significantly higher among mothers with preterm birth compared to those mothers with full term birth (p = 0.002). Similarly, the risk of preterm birth was significantly higher among mothers with hypertension (p = 0.002) and mothers with vaginal infections (p = 0.029).

**Table 3:** Gynecological factors associated with preterm birth

Attributes	Pre-term		Full-term		Total		p value
	n	%	N	%	n	%	
**Hospitalization during pregnancy**							
Yes	8	7.1	14	2.0	22	2.7	**0.002**
No	105	92.9	690	98.0	795	97.3	
**Any chronic diseases**							
Yes	5	4.4	25	3.6	30	3.7	0.647
No	108	95.6	679	96.4	787	96.3	
**Gestational Diabetes**							
Yes	0	0.0	1	0.1	1	0.1	0.689
No	113	100.0	703	99.9	816	99.9	
**Hypertension during pregnancy**							
Yes	5	4.4	6	0.9	11	1.3	**0.002**
No	108	95.6	698	99.1	806	98.7	
**Malaria during pregnancy**							
Yes	1	0.9	5	0.7	6	0.7	0.840
No	112	99.1	699	99.3	811	99.3	
**Hepatitis B**							
Yes	0	0.0	1	0.1	1	0.1	0.689
No	113	100.0	703	99.9	816	99.9	
**Urethritis infection**							
Yes	7	6.2	51	7.2	58	7.1	0.687
No	106	93.8	653	92.8	759	92.9	
**Cystitis infection**							
Yes	24	21.2	135	19.2	159	19.5	0.607
No	89	78.8	569	80.8	658	80.5	
**Pyelonephritis infection**							
Yes	5	4.4	40	5.7	45	5.5	0.587
No	108	95.6	664	94.3	772	94.5	
**Vaginitis infection**							
Yes	18	15.9	65	9.2	83	10.2	**0.029**
No	95	84.1	639	90.8	734	89.8	

### Bivariate and multivariable logistic regression analysis of factors predicting preterm birth

On bivariate logistic regression analysis, maternal age, secondhand smoking, ANC visits, history of abortion, preterm rupture of membranes, hospitalization, hypertension, and vaginal infections were significantly associated with preterm delivery at a p value less than 0.05. After considering all these variables together in multivariable logistic regression by specifying the backward conditional method, maternal age, second-hand smoking, history of abortion, preterm rupture of membrane, and hypertension were predicting preterm birth (Table 4).

**Table 4:** Bivariate and multivariable logistic regression analysis of factors predicting preterm birth

Variables	Unadjusted OR (95%CI)	Adjusted OR (95%CI)
**Maternal age [years]**		
15 to 24	1.00	1.00
25 to 34	1.23(0.78–1.93)	1.27(0.79–2.03)
35 to 49	**2.14(1.25–3.65)**	**2.00(1.13–3.53)**
**Partner’s smoking status**		
Yes	**2.01(1.12–3.60)**	**1.91(1.04–3.51)**
No	1.00	1.00
**ANC visits**		
< 4	1.63(1.08–2.46)	1.51(0.98–2.34)
>=4	1.00	1.00
**History of abortion**		
Yes	**1.80(1.10–2.95)**	**1.89(1.13–3.15)**
No	1.00	1.00
**Preterm rupture of membrane**		
Yes	**11.29(4.02–31.73)**	**9.30(3.18–27.16)**
No	1.00	1.00
**Hospitalization during pregnancy**		
Yes	**3.75(1.54–9.17)**	**2.23(0.75–6.60)**
No	1.00	1.00
**Hypertension in Pregnancy**		
Yes	**5.39(1.62–17.95)**	**4.40(1.18–16.42)**
No	1.00	1.00
**Vaginal infection**		
Yes	**1.86(1.06–3.28)**	1.39(0.75–2.57)
No	1.00	1.00

Older mothers aged 35 to 49 years were 2 times more likely to deliver preterm babies compared to younger mothers aged 15 to 24 years (AOR = 2.00; 95%CI = 1.13–3.53). Preterm birth was 1.9 times more likely to occur among mothers exposed to secondhand smoke (AOR = 1.91; 95% CI = 1.04–3.51). Mothers who had previously had an abortion had a 1.9-fold increased risk of preterm birth (AOR = 1.89; 95% CI = 1.13–3.15; Preterm birth was about nine fold more common among mothers who experienced preterm membrane rupture (AOR = 9.30; 95CI = 3.18–27.16) and 4.4 times more common among hypertensive mothers (AOR = 4.40; 95% CI = 1.18–16.42).

## Discussion

The purpose of this study was to establish maternal, obstetric, and gynecological factors associated with preterm birth in Rwanda. In our study, the prevalence of preterm birth was found to be 13.8%. After adjusting for potential confounders using a multivariable logistic regression model, the identified independent risk factors associated with preterm birth were advanced pregnancy age, secondhand smoke, abortion history, preterm membrane rupture, and pregnancy-induced hypertension.

The prevalence of preterm birth in Rwanda was higher than studies done in multi-center health facilities in Iran (5.1%) [28] and in Australia (6.8%) [29], as well as the WHO estimates for sub-Saharan Africa (9.5%) and lower-income countries (12.0%) [30]. However, it was similar to the pooled prevalence from metal-analysis in Ethiopia (11.4%) [31] as well as findings from multi-center study in Brazil (12.3%) [32] and from a referral hospital in Tanzania (14.2%) [33]. Further, it was slightly lower than a study conducted in Kenya (18.3%) [6] and in one District Hospital in Rwanda (17.5%) [26].

However, this prevalence is lower than recent cross-sectional studies done in teaching hospitals in Ghana, whereby it was reported at 37.3% [34], and 25.9% in Ethiopia [10]. These disparities in the prevalence of preterm birth among the studies could be attributed to small sample sizes in a small-scale study setting [9, 26, 34]. It could also be the method used to estimate the gestational age, as most studies depend on the last menstrual period reported retrospectively by the mothers, which could result in a biased prevalence [6, 26, 35]. Moreover, most studies recruited respondents from a single hospital, which could possibly overestimate the prevalence of preterm birth [9, 34, 36]. Our study, on the other hand, used a sufficient sample size from the community and the gold standard approach of ultrasonography to determine gestational age.

Our study showed that advanced maternal age was associated with preterm birth. Several studies have also found that advanced maternal age is a risk factor for preterm birth [16, 17, 37–39]. This increased risk of preterm birth among women of advanced age (> 35 years) could be attributed to the fact that reproductive organs and fertility decrease after 35 years. Moreover, complications related to pregnancy are more common in this advanced age group. However, other research from the United Kingdom [40] failed to detect an association, which may be related to socio-economic differences.

It is acknowledged that the primary risk factor for preterm birth is premature membrane rupture [41]. According to our research, women who experienced premature membrane rupture had a 9.3 times higher risk of having a preterm birth. This is consistent with several other studies [6, 36, 42–46]. This may be as the result of endogenous prostaglandins released during membrane rupture, which start the uterine contraction and lead to preterm birth [31]. This implies that pregnant women who have a history of PROM should receive additional care and management.

In concordance with other previous studies, [34, 43, 47, 48], this current study observed that hypertension during pregnancy was an independent risk factor for preterm birth. In East Africa, studies conducted in Kenya and Tanzania found that pregnancy induced hypertension is a risk factor for preterm birth [6, 33]. The poor pregnancy outcomes linked to hypertensive diseases during pregnancy, including premature delivery, are plausibly explained by uteroplacental ischemia [49, 50], despite the biology of this illness still not being fully understood. Thus, early detection of pregnancy-induced hypertension during antenatal care visits and screening is critical for appropriate treatment and management.

The current study found that women with a history of abortion were significantly more likely to give birth prematurely. This is in agreement with other studies as well as a systematic review and a meta-analysis [9, 10, 45, 51, 52]. According to the literature, the risk of abortion in early pregnancy is associated with undesirable birth outcomes, including preterm birth. The risk of infection associated with recurrent abortion may be the biological mechanism causing this connection. It has been reported that women who have had abortions before are more likely to get intra-amniotic infections [53]. Preterm delivery has a known risk factor known as intra-amniotic infection [54]. Women and medical professionals (midwives and obstetricians) should also be made aware of the potential link between abortion and premature delivery.

Our study also revealed that pregnant women exposed to secondhand smoking had significantly higher odds of delivering premature babies. There has been evidence that pregnant women who smoke cigarettes are at increased risk of preterm birth [9, 10, 29, 55]. In our study, the number of women who smoked during pregnancy was not significant (p = 0.089). However, the link between secondhand smoke exposure and preterm birth is still up for debate. Two recent studies done in Vietnam and the USA depicted a significant association between preterm birth and secondhand smoking [56, 57]. The pathway and connection between secondhand smoke and preterm delivery must be better understood in order to guide policymakers in putting preventative initiatives in place.

Although UTI and ANC attendance were significant in bivariate analysis, they were not significant in multivariate analysis. This finding is similar to that of a study conducted in Kenya which reported no significant association between preterm birth and number of ANC visits [6]. In contrast, a systematic and meta-analyses study found that women who had less than four ANC visits had a higher risk of preterm birth [45], which could be due to a failure to identify preterm birth risk factors during ANC visits. Other studies and reviews have found that maternal urinary tract infection (UTI) is linked to preterm birth [6, 20, 45], with infections weakening the baby’s amniotic sac, leading to PROM and preterm birth [58].

One of the study’s strengths is its large representative sample drawn from 30 health facilities selected in 10 districts. The findings thus represent the prevalence of preterm births in Rwanda with reasonable certainty. This study does have some drawbacks, though. The many independent variables and preterm birth do not necessarily have a cause-and-effect connection. Additionally, there may be a chance of recollection bias because the data were obtained through interviews. Loss to follow-up (LTFU) among recruited persons was another constraint (24.97%), but after comparing the baseline characteristics of the LTFU and those of participants included in the data analysis, there were no significant discrepancies, ensuring the internal validity of the study.

## Conclusion

The magnitude of preterm birth was slightly higher than in the most recent Rwandan demographic health survey. It was independently affected by advanced age during pregnancy, secondhand smoke, a history of abortion, preterm membrane rupture, and pregnancy-induced hypertension. Therefore, it is preferable to take into account pregnant women who are older, exposed to secondhand smoke, have had abortions before, and have hypertension when screening and intervening in order to avoid the short- and long-term effects of premature birth.

## Data Availability

The data and materials used in this study are available from the corresponding author upon request.

## List of Abbreviations

AOR: Adjusted odds ratio
ANC: Antenatal care
COR: Crude odds ratio
HIV: Human Immunodeficiency Virus
LMP: Latest menstrual period
PROM: Premature membrane rupture
SPSS: Statistical package for social sciences
USA: United States of America
UTI: Urinary tract infection, WHO:World Health Organization
WHO: World Health Organization
